# The association between maternal hepatitis B e antigen status, as a proxy for perinatal transmission, and the risk of hepatitis B e antigenaemia in Gambian children

**DOI:** 10.1186/1471-2458-14-532

**Published:** 2014-05-30

**Authors:** Yusuke Shimakawa, Christian Bottomley, Ramou Njie, Maimuna Mendy

**Affiliations:** 1 Medical Research Council (MRC) Unit, Atlantic Boulevard, Fajara, P.O. Box 273, Banjul, The Gambia; 2 Department of Infectious Disease Epidemiology, London School of Hygiene and Tropical Medicine, Keppel Street, London WC1E 7HT, UK; 3 The Gambia Hepatitis Intervention Study, IARC, c/o MRC Unit, Atlantic Boulevard, Fajara, P.O. Box 273, Banjul, West Africa, The Gambia; 4 International Agency for Research on Cancer (IARC), 150 Cours Albert Thomas, Lyon, CEDEX 08 69372, France

**Keywords:** Hepatitis B, Hepatitis B e antigens, Infectious disease transmission, Vertical, Age factors, Africa

## Abstract

**Background:**

Early age at infection with hepatitis B virus (HBV) increases the risk of chronic HBV infection. In addition early age at infection may further increase the risk of persistent viral replication beyond its effect on chronicity. The effects of perinatal and early postnatal transmission on the risk of prolonged hepatitis B e antigenaemia in children with chronic HBV infection are not well documented in Africa. We examine these associations using maternal HBV sero-status and the number of HBV-positive older siblings as proxy measures for perinatal and early postnatal transmission, respectively.

**Methods:**

Hepatitis B e antigen (HBeAg)-positive mothers were identified in six population-based HBV sero-surveys conducted in The Gambia between 1986 and 1990. For every HBeAg-positive mother, a hepatitis B surface antigen (HBsAg)-positive HBeAg-negative mother and HBsAg-negative mother were randomly selected from the population surveyed. These mothers and their family members were tested for HBV sero-markers in a subsequent survey conducted between 1991 and 1993.

**Results:**

Thirty-eight HBeAg positive mothers and the same number of HBsAg-positive HBeAg-negative mothers and HBsAg-negative mothers participated in the study. Sixty-nine percent of their children also participated. There was a non-significant positive association between HBeAg prevalence in children and the number of HBeAg-positive older siblings (64.1%, 69.2% and 83.3% in children with 0, 1 and ≥2 HBeAg-positive older siblings, respectively). After adjusting for confounders, having an HBeAg-positive mother was a risk factor for HBeAg positivity in children carrying HBsAg (adjusted OR 4.5, 95% CI: 1.0-19.5, p = 0.04), whilst the number of HBeAg-positive older siblings was not.

**Conclusions:**

Maternal HBeAg was associated with positive HBeAg in children with chronic HBV infection. This suggests that interrupting mother-to-infant transmission in sub-Saharan Africa might help reduce the burden of liver disease. A timely dose of HBV vaccine within 24 hours of birth, as recommended by WHO, should be implemented in sub-Saharan Africa.

## Background

Chronic infection with the hepatitis B virus (HBV) is a cause of hepatocellular carcinoma (HCC) [1]. The risk of chronic infection after exposure to HBV depends on the age at infection; infection becomes chronic in 80–90%, 20–30%, <10%, and <5% of individuals who are infected during perinatal period, early childhood, adolescence and adulthood, respectively [2]. Early HBV infection is therefore associated with a higher risk of HCC through the increased risk of chronic infection [3]. However, beyond its effect of increasing the chance of becoming a chronic carrier, early age at HBV infection may further increase the risk of persistent viral replication, which ultimately leads to HCC [4]. The immaturity of the host immune system in neonates and toddlers has been suggested as a mechanism of prolonged e antigenaemia [5].

A recent systematic review of observational studies [6] found a positive association between having a mother positive for HBV sero-marker (a proxy for perinatal mother-to-infant transmission) and prolonged hepatitis B e antigenaemia (an indicator of high viral replication) amongst children with chronic HBV infection. There was also a positive association between maternal sero-positivity and paediatric HCC. These findings suggest that HBV infection in early life through perinatal maternal transmission might increase the risk of HCC by maintaining a high viral replication.

However, the scope of the review was limited since: 1) most of the studies included were from East Asia where mother-to-infant transmission is frequent [7], and 2) none of the studies examined the infectious status of older siblings, who are known to be a major source of HBV infection in many parts of the world including sub-Saharan Africa (sSA) [5,8,9]. Thus, the contribution of sibling-to-sibling HBV transmission during early childhood to the prolonged viral replication remains to be determined.

In this study we examined the association between age at HBV infection and the presence of hepatitis B e antigen (HBeAg) in The Gambia, West Africa, using maternal HBV sero-status and number of HBV-positive older siblings as a proxy for perinatal and early postnatal transmission, respectively. The analysis was restricted to study participants with positive hepatitis B surface antigen (HBsAg), because our aim was to identify the effect of age at infection (or mode of transmission) on the risk of hepatitis B e antigenaemia, beyond its effect of increasing the risk of chronic HBV infection [6].

## Methods

### Setting

**Figure 1:**
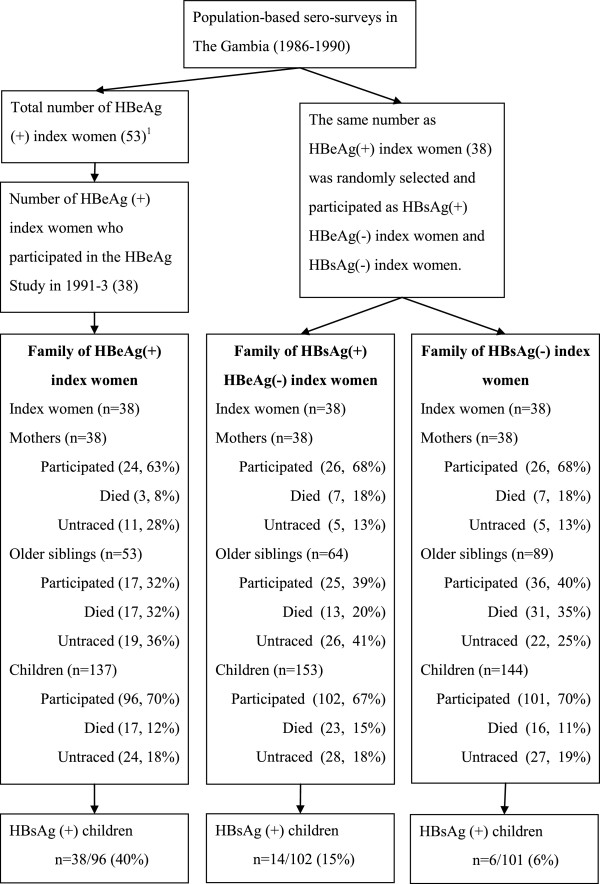
**Flow chart of study participants in the HBeAg Study, The Gambia, 1991–1993. **^1^Origins of HBeAg-positive mothers are the following: GHIS group 1 (n = 18), GHIS group 3 (n = 17), Arthropod study (n = 6), Manduar sero-survey (n = 4), Farafenni sero-survey (n = 6), and Banjul sero-survey (n = 2).

A nation-wide hepatitis B vaccination trial was initiated in The Gambia in 1986 and by 1990 countrywide coverage was achieved [10]. In parallel, six population-based sero-surveys for HBV infection were conducted in The Gambia between 1986 and 1990 to assess immunological response to the vaccine and to determine risk factors for HBV transmission [9-11]. These surveys obtained sera from both children and their mothers and tested for the presence of HBsAg in all the samples, and those that tested positive for HBsAg were further tested for HBeAg. In total, 53 HBeAg-positive mothers were identified from these studies (Figure 1).

On the basis of the HBV sero-status determined at these surveys, all HBeAg-positive mothers were invited to participate in “HBeAg Study”. The same numbers of HBsAg-positive HBeAg-negative mothers and HBsAg-negative mothers were randomly selected from the databases used in these surveys. Between 1991 and 1993, these mothers (i.e., index women) and their family members were bled for HBV markers to determine the effect of familial HBV sero-markers on presence of HBeAg.

After consent was obtained, the index women were bled and interviewed to collect demographic information on dead and living family members. Index women were bled twice, the first bleeding occurred between 1986 and 1990 and the second at the time of the HBeAg Study (1991–1993). We used the serological status recorded in the first survey as a proxy for serological status at birth of their children.

Family members of the index woman (excluding half siblings), were contacted and if they agreed to participate in the study, were bled for HBV serology. All sera were assayed for HBsAg by reverse passive haemagglutination (Wellcotest, Murex Diagnostics, UK). HBeAg was tested by radioimmunoassay (Sorin, Biomedica, Italy) only when sera were HBsAg-positive. The study was approved by the Gambia Government/MRC (Medical Research Council) Joint Ethical Committee and the ethics committee at IARC (International Agency for Research on Cancer), France.

### Data analysis

The association between maternal HBV markers (HBsAg, HBeAg) and e antigenaemia was examined in successive generations: mothers of index women (1^st^ generation) and index women (2^nd^ generation); and index women (2^nd^ generation) and their children (3^rd^ generation). The effect of the number of older siblings positive for HBsAg or HBeAg was evaluated in index women (2^nd^ generation) and their children (3^rd^ generation).

Logistic regression was used to estimate adjusted odds ratios for the associations between familial HBV sero-status (maternal sero-status and number of elder siblings positive for HBV markers) and e antigenaemia in index women (2^nd^ generation). In the children of index women (3^rd^ generation), adjusted odds ratios for the same associations were estimated using generalised estimating equations with an exchangeable correlation structure to account for household clustering (the previous analysis did not account for household clustering because none of index women shared the same mother). A linear test for trend was used to assess the statistical significance of the number of elder siblings positive for HBeAg and the number positive for HBsAg.

**Figure 2:**
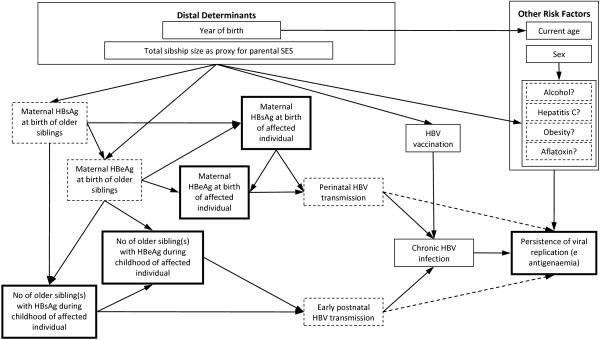
**Causal diagram for the effect of familial HBV marker on hepatitis B e antigenaemia in the HBeAg Study, The Gambia, 1991–1993.** Maternal HBV sero-marker and number of older siblings with positive HBV sero-marker are proxies for perinatal and early postnatal HBV transmission, respectively. Early age at HBV infection is associated with positive HBeAg through increasing the risk of chronic HBV infection. The hypothesis of this analysis is in addition to this effect, early age at infection further increases the risk of hepatitis B e antigenaemia (presented as a dashed arrow). The variables which were not measured are surrounded by dashed lines, and the exposure and outcome variables of interest are surrounded by lines in bold-type. SES denotes socio-economic status.

A minimally sufficient set of *a priori* confounders of the association between each of the familial sero-markers and e antigenaemia was identified from a causal diagram (Figure 2) by applying the backdoor test [12]. The set of confounders was the same for each familial sero-marker, and consisted of: year of birth, total sibship size (as a proxy for parental socioeconomic status), and the other familial HBV sero-status measures. To estimate the effect of maternal HBeAg, the set of confounders was: year of birth, total sibship size, maternal HBsAg, number of older siblings with positive HBsAg, and number of older siblings with positive HBeAg. And to estimate the effect of the number of older siblings with positive HBeAg, the set of confounders was: year of birth, total sibship size, maternal HBsAg, maternal HBeAg, and number of older siblings with positive HBsAg. A history of hepatitis B vaccination in children of index women was not considered a confounder for the association of maternal HBV sero-status with e antigenaemia in children, because hepatitis B vaccine was given irrespective of maternal HBV sero-status [10,13]. The mothers of index women (1^st^ generation) were all negative for HBeAg and only one index woman had an HBeAg-positive older sibling. These variables were therefore omitted from multivariable models of e-antigenaemia in index women (2^nd^ generation).

## Results

### Participation

Out of 53 HBeAg-positive index women (2^nd^ generation) in the database, 38 (71.7%) were traced and agreed to participate in the study. The rate of refusal among HBeAg-negative index women was not recorded. More than 65% of the mothers (1^st^ generation) and children (3^rd^ generation) of index women participated in the study, but participation was less than 35% among older siblings of index women (Figure 1).

### Index women (2^nd^ generation)

The characteristics of 38 HBsAg-positive HBeAg-positive index women and 38 HBsAg-positive HBeAg-negative index women are presented in Table 1. The median age in index women was 25.0 years (interquartile range: 23–30) for HBeAg-positive and 26.5 years (interquartile range: 22–30) for HBeAg-negative index women (Wilcoxon rank-sum test: p = 0.75). The associations between familial HBV sero-status and HBeAg positivity in HBsAg-positive index women are presented in Table 2. The proportion of index women who have HBsAg-positive mothers was higher in HBeAg-positive index women (16.7%, 4/24) than in HBsAg-positive HBeAg-negative index women (7.7%, 2/26), although the difference was not statistically significant (p = 0.33). None of the HBsAg-positive mothers of index women (1^st^ generation) were HBeAg-positive. The proportion of index women with HBsAg-positive older siblings was similar in HBeAg-positive index women (15.4%, 4/26) and HBsAg-positive HBeAg-negative index women (17.2%, 5/29). After adjusting for confounding factors, there was no evidence for an association between HBeAg positivity in index women and either maternal HBsAg (OR 1.2, 95% CI: 0.1-23.2, p = 0.89) or having an HBsAg-positive older sibling (OR 0.9, 95% CI: 0.2-5.5, p = 0.92).

**Table 1:** **Characteristics of HBsAg-positive HBeAg-positive index women and HBsAg-positive HBeAg-negative index women (2**^**nd **^**generation)**

**Variables**		**HBsAg (+) HBeAg (+) index women (n = 38)**		**HBsAg (+) HBeAg (−) index women (n = 38)**	
		**No**	**%**	**No**	**%**
Age group	17-20	6	15.8	7	18.4
	21-30	25	65.8	22	57.9
	31-45	7	18.4	9	23.7
Year of birth	1948-1960	6	15.8	8	21.0
	1961-1970	27	71.0	24	63.2
	1971-1974	5	13.2	6	15.8
Total sibship size	1-4	16	42.1	8	21.0
	5-6	12	31.6	15	39.5
	≥7	10	26.3	15	39.5

**Table 2:** **Risk factors for HBeAg positivity in HBsAg-positive index women (2**^**nd **^**generation)**

**Variables**	**HBsAg (+) HBeAg (+) index women (n = 38)**		**HBsAg (+) HBeAg (−) index women (n = 38)**		**Crude odds ratios**			**Adjusted odds ratios**^**1**^		
	**No**	**%**	**No**	**%**	**OR**	**95% CI**	**P**	**OR**	**95% CI**	**P**
Maternal HBsAg (1^st^ generation)										
Negative	20	83.3	24	92.3	1.0		0.3	1.0		0.9
Positive	4	16.7	2	7.7	2.4	0.4-14.5		1.2	0.1-23.2	
Maternal HBeAg (1^st^ generation)										
Negative	24	100	26	100	1.0			1.0		
Positive	0	0	0	0	N/A			N/A		
No. of older siblings with positive HBsAg										
0	22	84.6	24	82.8	1.0		0.9	1.0		0.9
1-2	4	15.4	5	17.2	0.9	0.2-3.7		0.9	0.2-5.5	
No. of older siblings with positive HBeAg										
0	25	96.2	29	100	1.0			1.0		
1	1	3.8	0	0	N/A			N/A		

^1^Model included maternal HBsAg, number of older siblings with HBsAg, year of birth, and total sibship size.

### Children of index women (3^rd^ generation)

In total, 96, 102 and 101 children of HBeAg-positive, HBsAg-positive and HBeAg-negative, and HBsAg-negative index women participated, respectively. Of whom, 38 (39.6%), 14 (13.7%) and 6 (5.9%) tested positive for HBsAg, respectively (p < 0.001, Figure 1). The characteristics of these HBsAg-positive children (n = 58) by maternal HBV marker are presented in Table 3. The median age was similar in the three groups. However, HBsAg-positive children with HBeAg-positive mothers were more likely to be female, HBV vaccinated and to have three or fewer siblings than HBsAg-positive children born to HBeAg-negative mothers.

**Table 3:** **Characteristics of HBsAg-positive children (3**^**rd **^**generation) according to maternal HBV sero-status**

**Variables**		**HBsAg(+) HBeAg(+) mother (n = 38)**		**HBsAg(+) HBeAg(−) mother (n = 14)**		**HBsAg(−) mother (n = 6)**	
		**No.**	**%**	**No.**	**%**	**No.**	**%**
Sex	Male	12	31.6	8	57.1	5	83.3
	Female	26	68.4	6	42.9	1	16.7
Age group	0-5	15	39.5	2	14.3	3	50.0
	6-10	16	42.1	9	64.3	2	33.3
	≥11	7	18.4	3	21.4	1	16.7
Year of birth	1973-1980	5	13.2	3	21.4	1	16.7
	1981-1985	18	47.4	9	64.3	3	50.0
	1986-1991	15	39.4	2	14.3	2	33.3
Total sibship size	1-3	10	26.3	1	7.1	1	16.7
	4-5	9	23.7	7	50.0	5	83.3
	6-7	19	50.0	6	42.9	0	0.0
HBV vaccination	No	28	73.7	14	100	6	100
	Yes	10	26.3	0	0.0	0	0.0

The associations between familial HBV sero-status and HBeAg positivity in HBsAg-positive children (3^rd^ generation) are presented in Table 4. There was a non-significant trend that HBeAg prevalence in children increased with increasing number of HBeAg-positive older siblings (64.1%, 69.2% and 83.3% in children with 0, 1, and ≥2 HBeAg-positive older siblings, respectively), but there was no trend after adjusting for confounding. In contrast, the presence of maternal HBeAg was a risk factor for HBeAg positivity after adjusting for confounding (adjusted OR 4.5, 95% CI: 1.0-19.5, p = 0.04). The prevalence of e antigenaemia was similar between vaccinated (70.0%, 7/10) and unvaccinated (66.7%, 32/48, p = 0.84) children.

**Table 4:** **Risk factors for HBeAg positivity in HBsAg-positive children (3**^**rd **^**generation)**

**Variables**	**Prevalence of HBeAg (+)**	**Crude odds ratios**			**Adjusted odds ratios**^**2,3**^		
		**OR**	**95% CI**	**P**	**OR**	**95% CI**	**P**
Maternal HBsAg (2^nd^ generation)							
Negative	83.3% (5/6)	1.0		0.5	1.0		0.5
Positive	65.4% (34/52)	0.4	0.1-4.2		0.4	0.1-5.3	
Maternal HBeAg (2^nd^ generation)							
Negative	60.0% (12/20)	1.0		0.5	1.0		0.04
Positive	71.1% (27/38)	1.6	0.4-6.1		4.5	1.0-19.5	
No of older siblings with positive HBsAg							
0	65.6% (21/32)	1.0		0.7^1^	1.0		0.9^1^
1	66.7% (10/15)	1.0	0.3-3.8		1.1	0.2-7.2	
≥2	72.7% (8/11)	1.4	0.3-6.4		1.2	0.0-47.5	
No. of older siblings with positive HBeAg							
0	64.1% (25/39)	1.0		0.4^1^	1.0		0.8^1^
1	69.2% (9/13)	1.3	0.3-4.9		1.1	0.3-4.9	
≥2	83.3% (5/6)	2.8	0.3-27.7		2.0	0.1-42.7	

^1^Linear test for trend.

^2^Model included maternal HBsAg, maternal HBeAg, number of older siblings with HBsAg, number of older siblings with HBeAg, year of birth, and total sibship size.

^3^Wald test from generalised estimating equations.

## Discussion

We examined the effect of HBV sero-markers in mothers and older siblings on hepatitis B e antigenaemia in children carrying HBsAg (3^rd^ generation). We found that e antigenaemia in children was positively associated with maternal HBeAg but not with maternal HBsAg. Likewise, the prevalence of e antigenaemia in children was higher if older siblings were HBeAg positive, although this result was not statistically significant. These relationships imply that the early establishment of chronic HBV infection through perinatal transmission from infectious mothers, as well as early postnatal transmission from infectious older siblings is associated with presence of HBeAg. In addition, this is consistent with the observation that positive HBeAg confers greater infectivity than HBsAg positivity alone. For example, the risk of perinatal transmission is 10–17% in HBsAg-positive mothers without HBeAg and 63–67% in HBeAg positive mothers in sSA [14,15]. Indeed, in the current study, we observed that prevalence of HBsAg in children with HBeAg-positive mothers was significantly higher than that in children with HBsAg-positive but HBeAg-negative mothers (40% versus 15%).

The associations of familial HBeAg with e antigenaemia in index women (2^nd^ generation) could not be confirmed because all of the mothers of index women (1^st^ generation) tested negative for HBeAg and only one older sibling of index women tested positive for HBeAg. The low prevalence of HBeAg in the mothers and older siblings of index women was expected since HBeAg is lost over time. In The Gambia, 85% of children who established chronic HBV infection during early childhood had lost HBeAg by the second decade of life [16]. This makes it difficult to investigate the effect of familial HBeAg status in adults.

A strength of our study was that we could assess the effect of familial HBV sero-status, rather than a family history of HBV infection. The latter is inaccurate because the absence of such a history does not necessarily indicate that the family member was sero-negative for HBsAg [6]. Second, we could control for the HBV markers of older siblings when assessing the association of maternal HBV sero-status with HBeAg positivity. And similarly the association between siblings’ sero-status and HBeAg positivity was adjusted for maternal HBV markers. In other studies this has not been possible [4,17-22]. The mutual adjustment with siblings’ sero-markers was particularly relevant in The Gambia, where sibling-to-sibling transmission during early childhood is the most frequent route [8,9]. After adjustment the OR for the association between maternal HBeAg and e antigenaemia in children increased whilst the ORs for the number of HBeAg-positive older siblings decreased. This suggests that perinatal maternal transmission might be more important in determining the risk of persistent viral replication than early horizontal transmission from older siblings in The Gambia. This is consistent with the hypothesis that earlier age at HBV infection is associated with higher risk of e antigenaemia.

Two African case–control studies have investigated the association between age at HBV infection and HCC, ultimate sequelae of chronic HBV infection. Larouze *et al.*, found that the prevalence of maternal HBsAg was higher in HCC cases (71.4%, 20/28) than in healthy controls (14.3%, 4/28, P < 0.0001) while the prevalence of HBsAg in siblings was similar in cases (9.6%, 7/73) and controls (14%, 8/58) [3]. Ryder *et al*., studied the association between birth order and HCC, and found strong evidence that higher birth order, and hence earlier age at infection, is associated with higher HCC risk (P < 0.005) [23]. In the absence of immunisation, children with low birth order are exposed to HBV after they start schooling, whilst children with high birth order are infected much earlier by their older siblings who got the infection outside the household [24]. However, because individuals negative for HBsAg were included in both studies, the associations might be due to an increased risk of chronic HBV infection related with early age at infection. The results of these studies are therefore not directly comparable to our findings.

The study has several limitations. Firstly, the sample size was small, and the associations were therefore poorly estimated. Secondly, many family members of the index women did not participate in the HBeAg study, and this might have led to selection bias. Thirdly, important confounding variables might have been omitted, in particular viral genotype. In Taiwan where genotype B (80%) and C (20%) predominate, genotype C was found to be associated with delayed HBeAg seroconversion [25] and also with more frequent mother-to-infant transmission [4]. In The Gambia, genotype E is predominant (75–95%) followed by A [26-28]. Although there are only few African studies relating viral genotypes to clinical outcomes [29], preliminary data from other West African countries (Nigeria, Cameroon and Mali) suggest that HBeAg prevalence might be higher in subjects infected with genotype E (89.8%, 35/39) than in those with genotype A (33.3%, 2/6) [30].

HBV vaccination programmes in Africa and Asia have been successful in preventing postnatal horizontal transmission of HBV, thereby reducing the prevalence of chronic HBV infection [31,32]. However, they have had a limited impact on perinatal mother-to-infant transmission, especially when the mothers are HBeAg-positive or highly viraemic [31,33]. In East Asia, where perinatal maternal transmission is common, HBV vaccine is frequently given within 24 hours of delivery (i.e. timely birth dose) [34,35]. By contrast a timely birth dose of HBV vaccine is rarely administered in Africa because logistical challenges seem to outweigh its potential impact due to the relatively low frequency of mother-to-infant transmission [36]. Despite a high coverage of hepatitis B vaccine, only six countries in sSA are undertaking the birth dose by the end of 2012 and The Gambia is one of them [37]. However, in two villages in rural Gambia where HBV vaccine efficacy was evaluated [31], only 2.8% (63/2173) of children who were vaccinated against HBV between 1984 and 2007 were given a dose within 24 hours of birth (unpublished data). This might be because in The Gambia, most children are born at home, and are usually not taken away from home in the first week [38].

## Conclusion

Our study suggests that the risk of prolonged e antigenaemia, an important predictor of HCC [39], is higher in individuals who perinatally established chronic infection through infectious mothers than in those infected with HBV by horizontal transmission. This implies that interrupting mother-to-infant transmission in sub-Saharan Africa might help reduce the burden of liver disease. A timely dose of HBV vaccine within 24 hours of birth, as recommended by WHO [40], needs to be implemented in sub-Saharan Africa.

## Abbreviations

HBeAg: Hepatitis B e antigen; HBsAg: Hepatitis B surface antigen; HBV: Hepatitis B virus; HCC: Hepatocellular carcinoma; IARC: International agency for research on cancer; MRC: Medical research council; OR: Odds ratio; sSA: Sub-Saharan Africa; WHO: World health organisation.

## Competing interests

The authors declare that they have no competing interests.

## Authors’ contributions

MM conducted the surveys, performed laboratory tests and helped to improve the manuscript. YS designed the analysis of the study, performed the statistical analysis and drafted the manuscript. CB supervised the statistical analysis and helped to improve the manuscript. RN supported the conduct of the study. All authors read and approved the final manuscript.

## Pre-publication history

The pre-publication history for this paper can be accessed here:

http://www.biomedcentral.com/1471-2458/14/532/prepub
